# Filtering Light-Emitting Diodes to Investigate Amber and Red Spectral Effects on Lettuce Growth

**DOI:** 10.3390/plants10061075

**Published:** 2021-05-27

**Authors:** Bo-Sen Wu, Sarah MacPherson, Mark Lefsrud

**Affiliations:** Department of Bioresource Engineering, McGill University, 21111 Lakeshore Road, Sainte-Anne-de-Bellevue, QC H9X 3V9, Canada; bo-sen.wu@mail.mcgill.ca (B.-S.W.); sarahanne.macpherson@mcgill.ca (S.M.)

**Keywords:** amber light, controlled environment agriculture, LED, morphology, plant growth, red light

## Abstract

Red and blue light are the principal wavelengths responsible for driving photosynthetic activity, yet amber light (595 nm) has the highest quantum efficiency and amber-rich high pressure sodium lamps result in superior or comparable plant performance. On this basis, we investigated how lettuce plant growth and photosynthetic activity were influenced by broad and narrow light spectra in the 590–630 nm range, by creating amber and red light-emitting diode (LED) spectra that are not commercially available. Four different light spectra were outfitted from existing LEDs using shortpass and notch filters: a double peak spectrum (595 and 655 nm; referred to as 595 + 655-nm light) that excluded 630-nm light, 595-nm, 613-nm, and 633-nm light emitting at an irradiance level of 50 W·m^−2^ (243–267 µmol·m^−2^·s^−1^). Shifting LED wavelengths from 595 nm to 633 nm and from 595 nm to 613 nm resulted in a biomass yield decrease of ~50% and ~80%, respectively. When 630-nm light is blocked, lettuce displayed expanded plant structures and the absence of purple pigmentation. This report presents a new and feasible approach to plant photobiology studies, by removing certain wavelengths to assess and investigate wavelength effect on plant growth and photosynthesis. Findings indicate that amber light is superior to red light for promoting photosynthetic activity and plant productivity, and this could set precedence for future work aimed at maximizing plant productivity in controlled environment agriculture.

## 1. Introduction

Plant lighting experiments show that red (600–700 nm) light plays a critical role in photosynthetic activity and plant development within the photosynthetically active radiation (PAR) spectrum (400–700 nm) [1,2]. Pioneering action spectrum and quantum yield studies using monochromatic light indicate that red light induces higher photosynthetic activity (~20–40%) than other wavelengths in the PAR spectrum for typical greenhouse crops [3,4]. McCree [4] further determined that the highest wavelength peak of the action spectrum was at 620 nm, with a shoulder at 670 nm in the red wavelength range. This study led to the use of red light-emitting diodes (LEDs) in plant lighting systems [2,5].

Since the first data using 660-nm LEDs for plant lighting were reported by Bula et al. [6], the effect of deep-red (650–690 nm) LED light has been explored and evaluated for plant development [7,8,9]. Deep-red LEDs are still the basal component in plant LED lighting systems for plant productivity, since they are the most efficient LED light source [2,10,11]. Studies using 640-nm LEDs alone or as supplemental lighting to investigate plant responses did not report any positive effects on plant growth. However, 640-nm LED light stimulated secondary metabolite and anthocyanin accumulation [9,12,13,14]. To date, the effect of orange/red (610–630 nm) LED light, which McCree [4] reported as more effective wavelength at inducing higher photosynthetic activity, has not been clarified for typical greenhouse crops.

Amber-biased (~590–610 nm) high pressure sodium (HPS) lamps were the preferred choice over LEDs in commercial greenhouse facilities until recently, as plant productivity varies with respect to crop choice and growth stages when grown under LED light [13,15]. Experiments that compare HPS lamps to blue/red LEDs for plant growth and yield are a major focus of plant lighting studies [15,16,17,18,19]. LEDs have emerged as the prominent plant lighting system over HPS, mainly because of their higher energy efficiency. According to some of the same reports, plant productivity and physiology showed either no significant differences [15] or were superior when grown under HPS lamps alone [7,16,19]. In addition, conflicting results were reported on the effect of amber light using HPS lamps [20,21]. Specifically, suppressed growth of some greenhouse crops, including basil (*Ocimum basilicum*) and lettuce (*Lactuca sativa*, cv. Grand Rapids), was observed when grown under high proportions of amber light [20].

When compared to HPS lamps and other conventional lighting sources, LEDs are advantageous because specific wavelengths may be selected and controlled [5,22,23]. LED wavelengths can be selected to target specific plant physiobiological responses [13,24]. However, diode manufacturers offer limited options in terms of wavelength selection [25]. For instance, there are only 10–15 LED nominal wavelength options available in the red wavelength range from Cree and Philips Lumileds. Furthermore, users have limited control over existing LED light conditions (peak wavelength, spectral composition, and full width at half maximum [FWHM]) under normal operations. Although the lighting environment can be manipulated using different colored LEDs, this method only allows for combinations of existing LED colors. This has led to some undesirable outcomes, such as uneven light quality/quantity over plant surfaces and low light outputs in wavelengths of interest [26]. These limitations hinder investigations into the effect of specific narrow band wavelengths of LED light on plant growth and development.

Both the wavelength and efficiency of a lighting system determine plant growth and development. However, a knowledge gap and contradictory reported data exist regarding the 590 nm to 630 nm wavelength range. Users have limited access to LEDs with uncommon wavelengths so they cannot explore specific effects on plant photobiology. To address these challenges, the objectives of this study were to: (1) create single and double-peak LED spectra using optical filters that are not available from major LED manufacturers; (2) investigate the effects of 590–630 nm light from the spectra created with optical filters on photosynthetic activity and plant development in lettuce using LEDs at a high irradiance level. 

## 2. Materials and Methods

### 2.1. Plant Materials

Lettuce seeds (*L. sativa* cv. Breen; pelleted MT0 OG, Johnny’s Selected Seeds, Winslow, ME, USA) were potted in 25 mm rockwool growing cubes (Grodan A/S, Dk-2640, Hedehusene, Denmark) and placed in a germination chamber (TC30, Conviron, Winnipeg, Manitoba, ON, Canada) for germination. Plants were kept in the germination chamber under cool-white fluorescent bulbs (4200 K, F72T8CW, Osram, Wilmington, MA, USA) at an average irradiance level of 20 W·m^−2^ (equal to ~100 µmol·m^−2^·s^−1^) with a 16-h photoperiod. Environmental conditions in the chamber were controlled at 50% relative humidity, with day/night temperatures of 23/21 ± 1 °C and ambient CO_2_ levels. A 1× Hoagland nutrient solution was provided to plants every other day as described by Hoagland and Arnon [27]. The young lettuce plants (2 weeks after germination, in the germination chamber) were subjected to either whole plant photosynthetic rate measurements in the lab or light treatments in a growth chamber with the experimental LED lighting system.

### 2.2. LED Lighting System

**Figure 1:**
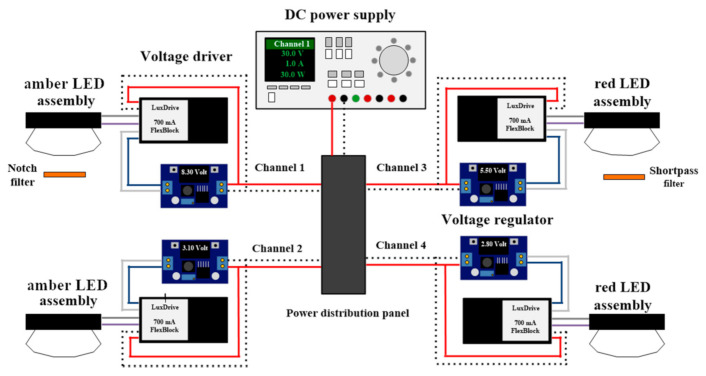
Simplified schematic diagram of the LED lighting system showing the power supply, power distribution panel, voltage drivers, voltage regulators, and LED assemblies.

Figure 1 illustrates the experimental LED lighting system designed for this study. Amber (LXM2-PL01, Philips Lumileds, San José, CA, USA) and red (LXM2-PD01, Philips Lumileds) LED assemblies were used as light sources. The amber LED assembly was selected because its spectral composition was similar to that of HPS lamps. Each LED assembly was connected to a power distribution panel and powered using a DC power supply (DP832, Rigol Technologies Inc., Beaverton, OR, USA). An adjustable voltage regulator with a digital voltmeter and a 700-mA dimmable DC voltage driver (A011 FlexBlock, LED dynamics, Randolph, VT, USA) was placed between the power distribution panel and the LED assembly; this provided a constant current output while allowing for adjustments to LED light outputs. All LED assemblies were mounted on a water jacket (ST-011, Guangzhou Rantion Trading Co., Guangdong, China) and attached to lenses (25 mm focal length, No. 263, Polymer Optics, Wokingham, Berkshire, UK) that concentrated emissions from the LED assemblies into one single spot (12 mm diameter). Water was circulated at 15 °C in the water jacket behind the mounted LEDs using an isotemp bath circulator (4100R20, Fisher Scientific, Hampton, NH, USA). Two types of optical filters were used to manipulate spectral compositions of the amber and red LEDs: a 632.8-nm notch filter (25 mm diameter, #67-120, Edmund Optics, Barrington, NJ, USA) and a 625-nm short pass filter (25 mm in diameter, #64-604, Edmund Optics).

### 2.3. Whole Plant Photosynthetic Rate Analyses

Whole plant photosynthetic rate analyses of two-week old lettuce plants were carried out using a LI-6400 Portable Photosynthesis System (LI-COR, Lincoln, NE, USA) equipped with a Whole Plant Arabidopsis Chamber (6400-17, LI-COR), in which different light treatments were provided by the external LED light sources (amber LED, amber LED+notch filter, red LED, and red LED+short pass filter), as described above. The irradiance level was set to 50 W·m^−2^ [equivalent to ~250 µmol·m^−2^·s^−1^ photosynthetic photon flux density (PPFD)]. Relative humidity and temperature of the LI-COR environment was controlled at 50% and 23 °C, the same as in the germination growth chamber. The CO_2_ concentration and flowrate in the Whole Plant Arabidopsis Chamber were set to 400 µL·L^−1^ and 400 µL·min^−1^, respectively. Whole plant photosynthetic rate measurements were collected on three independent biological replicates for each lighting condition. The measurement of photosynthetic rates lasted 30 min to one hour in duration with 4 s per data acquisition period for each replicate. The photosynthetic rate stabilized after 20 min of light exposure. Plant leaf area was measured using ImageJ 1.48v software (Bethesda, MD, USA). Imagery acquired with ImageJ software was used to determine the whole plant photosynthetic rate on a per unit leaf area basis.

### 2.4. Experimental Setup

After emergence of the fourth true leaf (2 weeks after germination in the germination chamber), lettuce plants were immediately transplanted to a growth chamber with the LED lighting system. Plants were cultivated in 13-L hydroponic tanks (Rubbermaid, Atlanta, GA, USA) under the four following different LED light treatments at an irradiance level of 50 W·m^−2^ for two weeks: an amber LED assembly, an amber assembly with the notch filter, a red LED assembly, and a red LED assembly with the shortpass filter. Peak wavelengths, spectral compositions, and light intensities (irradiance levels, PPFD, yield photon flux (YPF) [28]) of the LED light treatments were measured using a spectroradiometer (PS-300, Apogee, Logan, UT, USA). In the chamber, optical filters were secured using three-screw adjustable ring mounts (#36-605, Edmund Optics) and placed 25 mm below the amber and red LED assemblies. Cardboard covered with black plastic sheets, with a hole at the center, was placed on the ring mounts to avoid unfiltered LED spectra from reaching the plants. Between each light environment, black sheets were used as a light barrier. Due to the high irradiance levels and need for uniform light distribution over the plant surface, only a single lettuce plant was placed under each light treatment. Five biological replicates were repeated under each light treatment. Heights between the LED lights and the top of plants were checked every three days, and the LED lights were adjusted accordingly to allow the plant canopy to be exposed to the same irradiance level throughout the growth period. Differences in irradiance levels under each light treatment over the growth period were less than 2%. Fresh 1× Hoagland solution was provided weekly. Oxygen in the hydroponic tanks was provided using air pumps (Marina 200, Rolf C. Hagen Inc., Baie d’Urfé, QC, Canada). Environmental conditions (relative humidity, day/night temperature, CO_2_ levels, and photoperiod) in the chamber with the LED treatments were the same as the germination chamber. A timer connected to the DC power supply controlled the 16-h photoperiod.

### 2.5. Biomass Yield and Growth Parameter Analysis

After growing the plants for two weeks under the different LED light treatments, they were harvested and sampled for biomass yield and morphological analyses, including shoot fresh mass, dry mass, and leaf area. Biomass yield (fresh and dry mass) was determined with a balance (APX-153, Denver Instruments, Bohemia, NY, USA). To determine dry mass, plant samples were dried at a temperature of 75 °C for no less than 72 h. All biomass yield and growth parameters, including shoot fresh mass, shoot dry mass, and leaf area, were measured and reported under each light treatment.

### 2.6. Statistical Analysis

Statistical analyses were performed using JMP 10 software (SAS, Cary, NC, USA). Tukey–Kramer’s HSD was used for multiple comparisons among spectral treatment means obtained from significant one-way analysis of variance (ANOVA) tests (*p* < 0.05).

## 3. Results

### 3.1. LED Spectra in the 590-nm to 630-nm Range Obtained with Optical Filters

**Figure 2:**
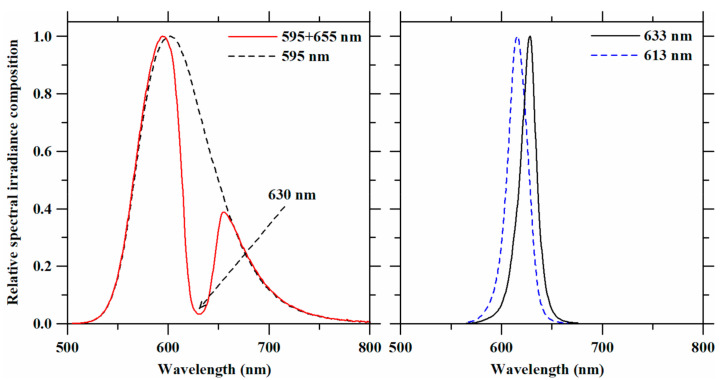
Relative spectral irradiance compositions of the LED spectra that were obtained with filters and used as light treatments on lettuce plants. The arrow in the upper figure indicates the wavelength of the valley using the amber LED assembly with the notch filter.

Shortpass and notch filters incorporated into the experimental LED lighting system resulted in single and double peak LED spectra (Figure 2) that made up the plant light treatments in this study. Peak wavelengths, spectral compositions, and light intensities of these light treatments are summarized in Table 1. Selection of the shortpass and notch filters was based on the requirement to decrease peak wavelengths of the red LED assemblies and to exclude overlapping wavelengths from other light treatments in the amber LED spectrum, respectively. Using the 625-nm short pass filter shifted the peak wavelength of 633-nm LED to 613 nm. With the notch filter, the amber LED spectrum was altered to narrow the 595-nm and 655-nm spectra (PPFD ratio ≅ 3:1). Four spectra were investigated in the plant lighting experiments: 595 nm (amber LED), 595 + 655 nm (amber LED with the notch filter), 613 nm (red LED with the short pass filter), and 633 nm (red LED). Although photosynthetic photon flux density (PPFD) is considered a standard unit for plant growth, it is usually used to define photosynthetic rates rather than photomorphogenesis. Therefore, light treatment intensity levels are reported as W·m^−2^, as recommended by Langhans and Tibbitts [29]. As we set the irradiance level at 50 W·m^−2^, the averaged light levels across the light treatments were 254 ± 5.08 µmol·m^−2^·s^−1^ (PPFD) and 244.5 ± 4.21 µmol·m^−2^·s^−1^ (YPFD).

### 3.2. Plant Growth and Morphology

**Figure 3:**
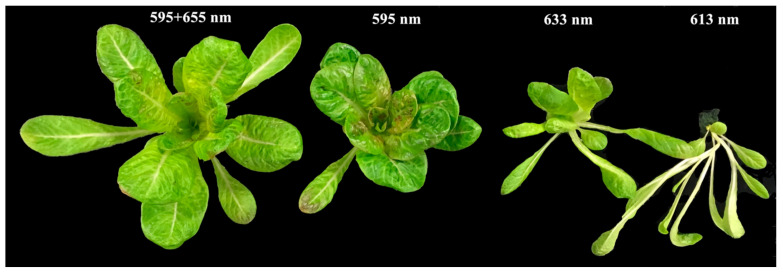
Plant morphology of lettuce (*Lactuca sativa*, cv. Breen) plants grown under 595 + 655-nm, 595-nm, 633-nm, and 613-nm light (from left to right) for two weeks. Wavelength values in the figure indicate the peak wavelengths of the light treatments.

**Figure 4:**
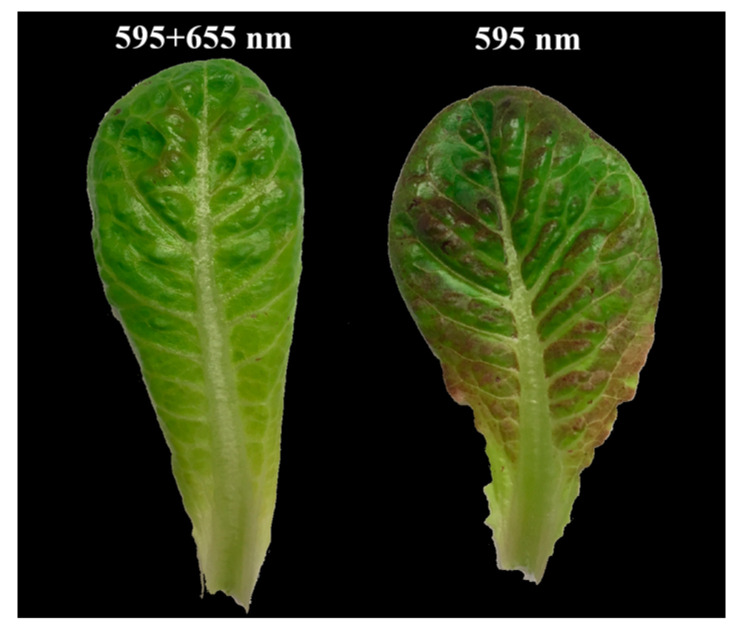
Representative pigmentation of a lettuce (*Lactuca sativa*, cv. Breen) leaf grown under 595 + 655-nm and 595-nm light treatments for two weeks.

To tease apart the effects of specific amber and orange/red wavelengths on plant photomorphogenesis and photosynthesis, hydroponically growing lettuce plants (*Lactuca sativa* cv. Breen) were treated with 595 + 655-nm, 595-nm, 633-, or 613-nm LED light spectra for two weeks. Lettuce plants exhibited the largest size when grown under the 595 + 655-nm light treatment, followed by the 595-, 633-, and 613-nm light treatments (Figure 3). Apparent leaf-elongated lettuce (poor leaf development) was observed under the 613- and 633-nm light treatments. Visible differences in leaf morphology and leaf coloration were observed across all light treatments. Lettuce plants grown under 595 + 655 nm and 595 nm light had more obvious lateral veins than those grown under the 613- and 633-nm light. Lettuce plants grown under the 595-nm light treatment were more compact than those grown under the 595 + 655-nm light treatment (Figure 3). Plants grown under the 613-nm light had longer and thinner leaves compared to the 633-nm light treatment. Lettuce leaves grown under 595 + 655-nm and 595-nm light exhibited more curliness near the lateral veins. However, they were relatively smooth when grown under the 613- and 633-nm light treatments. Interestingly, purple pigmentation was only observed for lettuce leaves grown under the 595-nm light treatment (Figure 4).

### 3.3. Photosynthetic Rates and Biomass Yield

Table 2 shows whole plant photosynthetic rates and biomass data for lettuce plants grown under 595 + 655-nm, 595-nm, 613-nm, and 633-nm light. Although the photosynthetic rate was similar among light treatments, the 613-nm light treatment induced the lowest photosynthetic rates and had 22% less photosynthetic activity than the 595-nm light treatment. Biomass yields (fresh and dry mass) and leaf area were generally higher under the broad-spectrum treatments (595 + 655- and 595-nm light treatments) than narrow-spectrum treatments (633- and 613-nm light treatments). All the experimental light treatments induced similar photosynthetic activities in lettuce, and differences in biomass yield between specific light treatments (i.e., 595-nm versus 613-nm treatments) were significant. For example, shoot fresh mass yield observed under the 595-nm light treatment (27.12 ± 2.68 g) was nearly fourfold higher than the lowest shoot fresh mass yield observed under the 613-nm light treatment (5.76 ± 1.75 g), whereas the difference in photosynthetic rate between the 595- and 613-nm light treatments was only ~20%. This large variation in dry mass yield was further observed between the 595- and 613-nm light treatments. It is important to note that shifting the LED wavelength from 595 to 633 nm, and from 595 to 613 nm, resulted in a biomass yield decrease (shoot fresh mass and dry mass) of approximately ~50 and ~80%, respectively.

## 4. Discussion

### 4.1. Photosynthetic Rate and Biomass Yield

Plant growth and development are products of plant photosynthesis and photomorphogenesis [4,30]. In this study, applying different light spectra did not greatly impact photosynthetic rate at ~250 µmol·m^−2^·s^−1^, yet it influenced plant development. Although light spectra with different bandwidths and intensities were used, similar photosynthetic rates between the 595-nm and 633-nm light treatments are consistent with previous findings on photon-weighed action spectrum [4,31]. A higher photosynthetic rate (~20%), however, was obtained under the 595-nm light treatment than the 613-nm light treatments. This large variation in photosynthetic activity is inconsistent with earlier PAR curve studies, as the relative quantum efficiency between 595 nm to 630 nm was between 0.92 to 1 [3,4]. Although these PAR curve studies provide baseline information on spectral photosynthesis, they were constructed under a low wavelength resolution (20 nm to 25 nm) and photosynthetic rates between these two measurement wavelengths are unknown (i.e., from 600 nm to 625 nm). These data indicate that replicating these pioneering works with a higher wavelength resolution could provide important information, particularly with LEDs gaining widespread popularity in controlled environment agriculture.

Higher shoot fresh mass, shoot dry mass, and leaf areas (approximately 50–80% higher) were observed in lettuce plants grown under the 595- and 595 + 655-nm light treatments when compared to the 613-nm and 633-nm light treatments, all with similar irradiance levels. For the amber wavelengths (595- and 595 + 655-nm), similar plant productivity is reported using HPS lamps with and without sunlight for greenhouse grown tomato (*Solanum lycopersicum* ‘Komeett’ F_1_ and ‘Starbuck’ F_1_) [19] and lettuce (*L. sativa* var. *capitata*) plants [16]. However, suppressed growth of lettuce (*L. sativa*, cv. Grand Rapids) under HPS lamps is reportedly caused by a high proportion of amber light [20]. If we compare light intensities used in these studies, we can see that a maximum PPFD level threshold strongly influenced plant growth under amber light. Suppression of lettuce growth occurred with a high proportion of amber light with HPS lamps at 500 µmol·m^−2^·s^−1^ [20]. In contrast, studies reporting a positive effect on plant growth occurred at lower HPS light intensities (80–170 µmol·m^−2^·s^−1^) [16,19]. The light intensity used in this study fell between the light intensities reported in the aforementioned work, at ~250 µmol·m^−2^·s^−1^ (243−267 µmol·m^−2^·s^−1^). Therefore, when comparing data from these studies and the current investigation, data suggest that, within the amber wavelength range, plants respond differently to irradiance levels of amber light. Low irradiance levels of amber light result in higher plant productivity, and these data support those reported by Gajc-Wolska, Kowalczyk, Metera, Mazur, Bujalski and Hemka [19] and Martineau, Lefsrud, Naznin and Kopsell [16]. In contrast, high irradiance levels of amber light lead to suppressed plant growth and to defense or interference of primary metabolism [20,21].

### 4.2. The Impact of Light Wavelegnth and Bandwidth on Biomass Production

High photosynthetic activity was measured in lettuce under 613- and 633-nm light treatments, yet lettuce leaf elongation and poor leaf development were observed. This suggests that 613- and 633-nm LED light affected plant morphology in a way that is similar to that of deep-red light [32,33]. Furthermore, although the 613- and 633-nm light treatments induced photosynthetic rates approximately 20% less than those of the 595- and 595 + 655-nm light treatments, biomass yield decreased by approximately 50% and 80% when shifting peak wavelengths from 633 nm to 613 nm and from 595 nm to 613 nm, respectively. This indicates that lettuce growth is strongly influenced by peak wavelengths in these LED spectra at high irradiance levels. These data are not consistent with conclusions made by Cope et al. [34] and Johkan et al. [35], who found that wavelength has a much smaller effect on plant growth rates than light intensity. The lack of accordance with our findings might be due to differences in PPFD levels and wavelengths used in these studies. We examined wavelengths and light intensities between 595–633 nm at 240–260 µmol·m^−2^·s^−1^, whereas Cope, Snowden and Bugbee [34] and Johkan, Shoji, Goto, Hahida, and Yoshihara [35] reported using white light and 510–530 nm LED light spectra, between 200–500 µmol·m^−2^·s^−1^ and 100–300 µmol·m^−2^·s^−1^, respectively.

Another reason for low correlation between photosynthetic activity and plant development with 613- and 633-nm light treatments could be interactive effects between plant architecture and photosynthetic activity. Because of instrument and chamber size limitations, two-week old plants were used for whole plant photosynthetic rate analyses. As plants grow, different photosynthetic rates are induced, and plant architecture is impacted. When plants are cultivated in a bigger environment, this results in different light interception efficiencies for leaves grown under different light treatments over the plant’s lifespan [36]. We observed considerable differences in biomass yield between different LED treatments, even though a constant irradiance level was provided over the plant canopies. This is exemplified by the lettuce plants grown under 595-nm and 613-nm light; the former wavelength induces higher photosynthetic rates and faster leaf expansion rates, resulting in different light interceptions despite having the same irradiance level over the plant canopies for both light treatments. Over time, differences in plant growth would become greater between plants, because of the total energy received by their leaves. Lastly, the leaf elongation induced by the 613-nm light treatment may have influenced light interception differences, resulting in the observed difference in biomass yield.

A low correlation between photosynthetic activity and plant development has been reported with narrow-spectrum amber light [4,37,38]. Narrow-spectrum amber light resulted in the highest photosynthetic activity in the PAR spectrum [4], yet induced lower leaf areas and stem elongation [37,38]. Non-photosynthetic wavelengths, the wavelengths having low pigment absorption, often leads to higher photosynthetic activity than the common blue and red wavelengths, mostly due to scattering effects that allow non-photosynthetic wavelengths to drive photosynthesis in the lower chloroplasts [39,40]. However, this narrow-spectrum amber light and the 613-nm light treatment used in this study cannot be sensed by photoreceptors that mediate and modulate structural plant development, resulting in poor development similar to shade-avoidance response [41,42]. While using the broad-spectrum amber light (595 + 655- and 595-nm light treatments), their spectrum ranges extend to the red and far-red wavelengths, eliciting shade-avoidance response via phytochromes [43]. Findings from this study provide a possible explanation as to why the low correlation between photosynthetic activity and plant development in narrow-spectrum amber light exists, and the motivation behind the recent popularity of white LED lights in plant lighting research [44,45]. 

### 4.3. Plant Architecture and Leaf Coloration

Blocking 630-nm light resulted in different leaf coloration and plant architecture when compared to the 595- and 595 + 655-nm light treatments. Sole or supplemental 650–660 nm light enhancing fresh/dry mass gain and leaf expansion has been observed in lettuce plants [32,46,47]. The effect of 630-nm light alone on growth or leaf expansion in lettuce has not yet been reported; however, it was explored in king protea (*Protea cynaroides*) [48], poinsettia (*Euphorbia pulcherrima*) [49], and cucumber (*Cucumis sativus* cv. Jinchun No. 4) [50]. When comparing lettuce plant morphology between the 595- and 633-nm light treatments, plants grown under the 595-nm light treatment displayed normal morphology with a compact architecture, while 613- and 633-nm light treatments prompted stem elongation with poor leaf development. Eliminating 630-nm light from the 595-nm light treatment resulted in plants with expanded structures when compared to the 595-nm light treatment. This suggests that the presence of 630-nm light impacts plant growth and morphology, unlike 650-nm light alone, which should not have any significant morphological impact [6]. These removing or adding individual wavelengths can alter plant architecture.

Purple coloration was observed in lettuce leaves grown under the 595-nm light treatment, but not when grown under 595 + 655-nm light, where in 630-nm light was blocked via the notch filter. Red and purple coloration in fruit and leaves is typically due to anthocyanin, a polyphenolic pigment [51]. Anthocyanin biosynthesis is often associated with high light conditions [52], as well as ultraviolet radiation and blue light [53,54]. In lettuce plants, anthocyanin accumulation can be induced by supplementing 373-, 455-, 460-, 476-, 505-, 658-, and 660-nm light with different light sources such as HPS lamps, solar light, and white fluorescent lamps [55,56,57]. However, anthocyanin accumulation mechanisms and interactions with light signal transduction pathways, particularly outside of blue wavelength range, are not yet understood [57]. In the present study, we observed that 630-nm light affects purple coloration in lettuce leaves. More validation with other greenhouse crops could clarify the effect of the 630-nm light on morphology and purple pigmentation in lettuce plants.

## 5. Conclusions

In summary, we investigated the influence of wavelength on lettuce plant growth and photosynthetic performance by filtering amber and orange/red light, yielding different spectra with the same irradiance level. Findings suggest that it is inappropriate to simply predict plant growth and development using photosynthetic data, and that the impact of wavelength on plant photomorphogenesis should be considered. This study further highlights the necessity of high controllability on amber and red light, as they may have positive or negative effects on photosynthetic activity, plant growth and development, and pigment accumulation. These data, coupled with previous studies, provide compelling evidence that amber light is superior to red light for promoting photosynthetic activity and plant productivity. To this end, our data add to our understanding of the impact of 590–630 nm light on both photosynthetic activity and plant development.

## Figures and Tables

**Table 1:** Peak wavelength, full width at half maximum (FWHM), irradiance level, photosynthetic photon flux density (PPFD), and yield photon flux density of each LED lighting system used in this study.

Light Treatments		Peak Wavelength (nm)	FWHM (nm)	Irradiance Level (W·m^−2^)	PPFD (µmol·m^−2^·s^−1^)	YPF (µmol·m^−2^·s^−1^)
595 + 655 nm	1st peak	595	48.90	50 (37.5 + 12.5)	250 (187.5 + 62.5)	240
	2nd peak	655	41.44			
595 nm		602	74.07	50	256	242
633 nm		633	20.24	50	267	257
613 nm		613	22.12	50	243	239

**Table 2:** Photosynthetic rates (*n* = 3) and biomass values (*n* = 5) (mean ± standard error) of lettuce (*Lactuca sativa*, cv. Breen) plants grown under different LED wavelengths. Letters ^a–h^ indicate significant differences (*p* ≤ 0.05).

LED Treatment	Photosynthetic Rate (μmol CO_2_ m^−2^ sec^−1^)	Shoot Fresh Mass (g)	Shoot Dry Mass (g)	Leaf Area (cm^2^)
595 + 655 nm	2.66 ± 0.16 ^ab^	27.16 ± 2.98 ^c^	1.16 ± 0.10 ^e^	419.5 ± 115.34 ^g^
595 nm	2.87 ± 0.08 ^a^	27.12 ± 2.68 ^c^	1.16 ± 0.11 ^e^	400.7 ± 66.25 ^g^
633 nm	2.34 ± 0.17 ^ab^	11.99 ± 1.54 ^d^	0.50 ± 0.08 ^f^	220.3 ± 45.13 ^h^
613 nm	2.31 ± 0.02 ^b^	5.76 ± 1.75 ^d^	0.25 ± 0.08 ^f^	136.6 ± 22.04 ^h^

## Data Availability

All data needed to evaluate conclusions in this study are presented in the text. Additional data related to this study are available from the corresponding authors upon reasonable request.
